# Variations in situational risk factors for fractures of the distal forearm, hip, and vertebrae in older women

**DOI:** 10.1186/s12877-021-02157-2

**Published:** 2021-03-31

**Authors:** Wen-Yu Yu, Hei-Fen Hwang, Mau-Roung Lin

**Affiliations:** 1 Department of Emergency Medicine, Taipei Medical University Hospital, Taipei, Taiwan, R.O.C.; 2 Institute of Injury Prevention and Control, College of Public Health, Taipei Medical University, 250 Wu-Hsing Street, Taipei, 11031 Taiwan, R.O.C.; 3 Department of Nursing, National Taipei University of Nursing and Health Sciences, Taipei, Taiwan, R.O.C.

**Keywords:** Fall, Fall prevention, Fracture, Situational factor, Injury, Older women

## Abstract

**Background:**

Situational factors during a fall among three common types of fractures of the distal forearm, hip, and vertebrae among older women in Taiwan were investigated.

**Methods:**

In 2016 ~ 2017, study participants were identified from those aged ≥65 years who visited emergency departments due to a fall in two university-affiliated hospitals in Taipei. In addition to individual characteristics, situational factors during the fall (location, activity, change of center of mass, fall mode, fall direction, initiating a protective response, and being hit) were collected. A sample of 203 distal-forearm fractures, 189 vertebral fractures, and 375 hip fractures was recruited, while 717 women with a soft-tissue injury were used as a control group. The identification of situational risk factors for each type of fracture was validated by using those who sustained one of the other two types of fracture as a control group.

**Results:**

After adjusting for age and other individual characteristics, compared to soft-tissue injuries, distal-forearm fractures were significantly more likely to occur with slips (odds ratio [OR] = 11.0; 95% confidence interval [CI] = 4.76 ~ 25.4), trips (OR = 3.40; 95% CI = 1.42 ~ 8.17), step-downs (OR = 4.95; 95% CI = 2.15 ~ 11.4), and from sideways falls (OR = 1.73; 95% CI = 1.12 ~ 2.67) and significantly less likely to occur indoors (OR = 0.62; 95% CI = 0.42 ~ 0.90) or from backwards falls (OR = 0.62; 95% CI = 0.41 ~ 0.95). Hip fractures were significantly more likely to occur with step-downs (OR = 1.76; 95% CI = 1.13 ~ 2.75) and from backwards (OR = 3.16; 95% CI = 2.15 ~ 4.64) or sideways falls (OR = 5.56; 95% CI = 3.67 ~ 8.41) and significantly less likely when hitting an object (OR = 0.26; 95% CI = 0.13 ~ 0.52) or initiating a protective response (OR = 0.58; 95% CI = 0.36 ~ 0.93). Vertebral fractures were significantly more likely to occur with slips (OR = 2.42; 95% CI = 1.30 ~ 4.50), step-downs (OR = 2.53; 95% CI = 1.43 ~ 4.48), and backwards falls (OR = 2.15; 95% CI = 1.39 ~ 3.32). Similar results were found in the validation analyses.

**Conclusions:**

Large variations in situational risk factors for the three types of fracture in older women existed. A combination of individual and situational risk factors may display a more-comprehensive risk profile for the three types of fracture, and an intervention that adds training programs on safe landing strategies and effective compensatory reactions may be valuable in preventing serious injuries due to a fall.

**Supplementary Information:**

The online version contains supplementary material available at 10.1186/s12877-021-02157-2.

## Background

Falls are a leading cause of injury and death in older adults. Approximately 5% ~ 10% of falls lead to major injuries such as fractures [1], and 90% of all fractures among people aged ≥65 years occur as a result of falls [2, 3]. Women are 50% more likely to have a fall injury and 75% more likely to have a lifetime fracture risk, compared to men [4, 5].

Although individual characteristics, such as age-related changes, a female gender, comorbidities, medications, and low bone mineral density (BMD), have been identified as risk factors for fractures [6], most of these individual characteristics are not modifiable or reliable for predicting fracture risks [1, 7]. On the other hand, situational factors during falls (e.g., circumstances and biomechanics of falls) were found to determine the type and severity of injury [8], and hence, identifying situational risk factors may help focus preventive efforts on falls with higher risks of fractures.

Among older women, the most common fractures are of the distal forearm, hip, and vertebrae [9]. While distal-forearm fractures occur most frequently as a result of a fall on an outstretched hand in healthy and active women with lower BMD [10], few studies have investigated the influence of situational exposures on the risk of other types of fracture, in which conflicting results exist as to whether a distal-forearm fracture is more likely to occur when falling backwards, sideways, or forwards [11–13]. A hip fracture is considered to be the most devastating consequence of falling in older people [14], and the risk of hip fractures may increase six-fold when falling sideways compared to falling backwards/forwards [15]. The vertebral body is the most common site of fractures, and an existing radiographic vertebral fracture also signals an increased risk of subsequent vertebral fractures and hip fractures [16, 17]. Nonetheless, no study has reported situational risk factors for vertebral fractures, although these factors could explain why most clinical vertebral fractures do not occur in older patients with a diagnosis of osteoporosis [18]. Comparisons of the relative impacts of situational factors on various types of fractures can provide information to more comprehensively describe risk profiles for the three types of fracture and for developing more-precise prevention programs for older people with various risks of a certain type of fracture.

Accordingly, a case-control study was conducted to investigate the effects of situational factors, in addition to individual characteristics, on the most common fractures of the distal forearm, hip, and vertebrae among older women in Taiwan.

## Methods

### Study participants

**Fig. 1:**
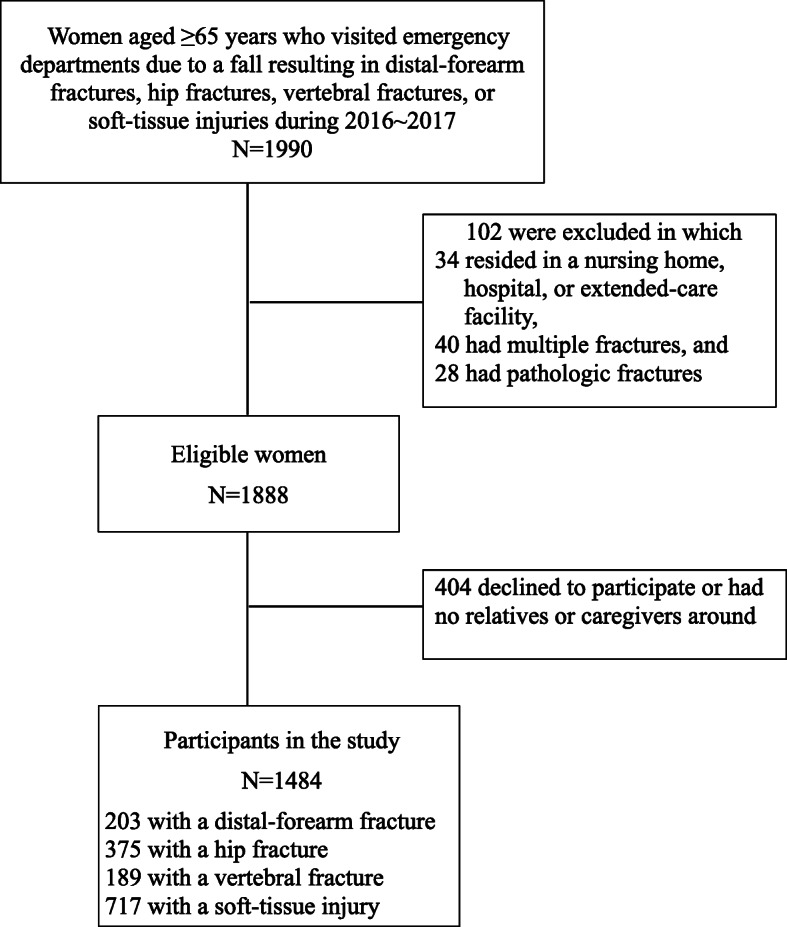
Flow chart of study participants

During a 2-year period in 2016 ~ 2017, eligible women were identified from those aged ≥65 years who had immediately visited the emergency department (ED) due to a fall resulting in a distal-forearm fracture, hip fracture, vertebral fracture, or soft-tissue injury of two university-affiliated hospitals in Taipei, Taiwan and who could ambulate within their own households prior to the fall. Those with only a soft-tissue injury of a sprain, strain, abrasion or contusion due to a fall occurring in the same time period were treated as a control group for the three fracture groups. Individuals were excluded if they resided in a nursing home, hospital, or extended-care facility at the time of the ED visit, or had multiple fractures or pathologic fractures caused by cancer, infection, inherited bone disorders, or a bone cyst. The progression of participants through the study is shown in Fig. 1. This research was reviewed and approved by the Institutional Review Board of Taipei Medical University, and written informed consent was obtained from each participant and main caregiver.

Each distal forearm or hip fracture was confirmed by radiography, a bone scan, or a **c**omputerized tomography (CT) scan. A diagnosis of clinical vertebral fracture was made from lateral spinal radiographs, according to Genant’s method based on the vertebral shape (wedge, biconcave, or compound), in which an illustrated atlas and a semiquantitative assessment of reductions in anterior, posterior, and middle vertebral heights were used [19], and a new vertebral fracture was diagnosed when patients had the presence of neck or back pain for the first time at the spinal site or the presence of bone marrow edema at the fracture site on magnetic resonance imaging (MRI) [20].

### Data collection

After a participant’s condition had stabilized during her/his stay in the ED observation unit or hospital ward, individual characteristics and situational factors were collected from medical records and through personal interviews with a structured questionnaire (provided in Supplementary Materials), and BMD measurements and functional tests were conducted. A main caregiver was interviewed when a subject was medically incapacitated (e.g., on ventilator support or comatose) or had difficulty communicating with the interviewers (e.g., severely cognitively impaired or with severe hearing loss).

### Individual characteristics

Individual characteristics were classified into three categories: sociodemographics/ lifestyles, medical characteristics, and functional abilities. Sociodemographics and lifestyles included the age at injury, educational level, living arrangement, body-mass index (BMI), cigarette smoking, alcohol consumption, and regular exercise habits. The BMI was calculated as the weight (kg) divided by the height squared (m^2^) and was categorized as being underweight (< 18.5 kg/m^2^), having an ideal weight (18.5 ~ 22.9 kg/m^2^), being overweight (23 ~ 24.9 kg/m^2^), and being obese (≥25 kg/m^2^) [21].

Medical characteristics included BMD measurements, fall history in the past year (none, 1, or ≥ 2 falls), fracture history since age 50 years (yes/no), number of chronic conditions, and medication use. The areal BMD of the left femoral neck was measured by dual-energy x-ray absorptiometry (DXA), using a Hologic Discovery Wi Bone Densitometer (Hologic, Bedford, MA, USA) and recorded as g/cm^2^ and a T-score. In participants who had undergone left hip replacement surgery, the right femoral neck was measured instead. Osteoporosis was defined as a BMD of 2.5 standard deviation (SD) units or more below the mean value for young women (T-score ≤ − 2.5) [22]. The number of chronic conditions was collected from a list of 12 conditions, including hypertension, heart disease, diabetes mellitus, stroke, respiratory tract disease, malignant tumors, gastric diseases, renal diseases, arthritis, cataracts, Alzheimer’s disease, and Parkinson’s disease. Medications consisted of antihypertensives, sedatives/hypnotics, antidiabetics, vitamins, calcium, and antihistamines.

Functional abilities, consisting of visual acuity, use of walking aids, cognitive status, depressive symptoms, and fear of falling, were assessed. Corrected visual acuity, tested by Rosenbaum cards, was categorized into good (≥20/50) and impaired (< 20/50) vision. The cognitive status was assessed using the 10-item Short Portable Mental Status Questionnaire (SPMSQ) and included the date, telephone number, street address, age, birthplace, maiden name, the current president, and digital subtraction (subtracting 3 from 20 sequentially, up to 6 times), and cognitive impairment was considered to be present when five or more errors occurred on the SPMSQ [23]. Depressive symptoms were tested using the 15-item Geriatric Depression Scale, with a score of > 5 being indicative of depression [24]. Fear of falling was measured using a 5-point Likert scale, with 1 being “not worried at all” and 5 being “extremely afraid”.

### Situational factors

Situational factors consisted of injury location (indoors or outdoors), activity during the fall (walking, toileting, getting in/out of bed/chair, negotiating stairs, doing housework, or others), change of center of mass (COM) (stable, vertical change, horizontal change, or both changes), mode (slipping, tripping, fainting, leg-weakness, or step-down), direction (forwards, backwards, or sideways), initiating a protective response (e.g., stepping, reaching, and grasping reactions), and hitting an object during the fall. For a COM change, activities of sitting, standing, and squatting were categorized as ‘stable’, those of getting up, standing up, sitting down, bending down, and jumping as ‘vertical change’, those of walking, turning, reaching, and running as ‘horizontal change’, and those involving both vertical and horizontal changes as ‘both changes’.

### Statistical analysis

Distribution patterns of individual characteristics and situational factors among the four study groups of distal-forearm fracture, hip fracture, vertebral fracture, and soft-tissue injury were compared using an analysis of variance (ANOVA) test for continuous variables, the Kruskal-Wallis test for ordinal variables, and Pearson’s Chi-squared test for categorical variables.

A multinomial logistic regression model was applied to investigate whether distributions of situational factors in each fracture group of the distal-forearm, hip, and vertebrae differed from those in the soft-tissue injury group, after adjusting for individual characteristics, and adjusted odds ratio (OR) and 95% confidence intervals (CIs) for these associations were estimated. The level of statistical significance for selecting variables in the final multivariable analysis was set to < 0.05. The goodness-of-fit of the model was tested using the Hosmer-Lemeshow test. For comparisons among different types of fracture, those variables which were statistically significant for one type of fracture were also retained for the other fracture types. To detect a statistical significance level of 0.05, the study sample of 203 patients with a distal-forearm fracture, 375 with a hip fracture, 189 with a vertebral fracture, and 717 patients with a soft-tissue injury provided a power of 72% ~ 98% for falling backward and of 62% ~ 100% for falling sideway vs. falling forward.

We also conducted binary logistic regression analyses to validate whether the selection and effect magnitude of situational risk factors for each type of fracture remained similar when patients who sustained the other two types of fracture, instead of those with a soft-tissue injury, were compared as the control group (e.g., when patients with a hip fracture were treated as cases, those with a distal forearm or vertebral fracture were classified into the control group). All statistical analyses were performed with the Statistical Analysis Software package vers. 9.4 (SAS Institute, Cary, NC, USA).

## Results

Of 1888 eligible women during the study period, 1484 agreed to participate in the study, among whom 203 had sustained a distal forearm fracture, 189 had sustained a vertebral fracture, 375 had sustained a hip fracture, 717 had sustained a soft-tissue injury, and 404 declined to participate or had no relatives or caregivers around to serve as proxies. Proxy respondents were obtained on behalf of 82 patients with a fracture and 39 patients with a soft-tissue injury. Participation rates of older women with distal-forearm fractures, hip fractures, vertebral fractures, and soft-tissue injuries were 83.1, 80.1,75.0, and 77.5%, respectively, and no significant differences in age (75.8 vs. 75.9 years; *p* = 0.960) or injury type (*p* = 0.102) between participants and non-participants were detected.

Distributions of sociodemographics and lifestyles, medical characteristics, and functional abilities among the four groups of distal-forearm fracture, hip fracture, vertebral fracture, and soft-tissue injury are shown in Table 1. Among the four groups, significant differences were found in the age at injury, educational level, living arrangement, BMI, regular alcohol consumption, regular exercise, BMD, fall history in the past year, presence of previous fractures since the age of 50 years, number of chronic conditions, medication use, use of sedatives/hypnotics and antidiabetics, visual acuity, use of walking aids, cognitive impairment, depressive symptoms, and fear of falling.

**Table 1:** Individual characteristics of older women with a distal-forearm fracture, vertebral fracture, hip fracture, or soft-tissue injury

Characteristic	Distal-forearm fracture (*N* = 203)	Hip fracture (*N* = 375)	Vertebral fracture (*N* = 189)	Soft-tissue injury (*N* = 717)	*p*
	*n* (%)	*n* (%)	*n* (%)	*n* (%)	
Age at injury (mean (SD)) (years)	72.0 (7.9)	79.9 (7.0)	76.3 (7.2)	75.5 (8.7)	< 0.001
Educational level					
Junior high or above	59 (29.1)	50 (13.3)	32 (16.9)	190 (26.5)	< 0.001
Elementary school	114 (56.1)	194 (51.8)	97 (51.3)	326 (45.5)	
Illiterate	30 (14.8)	131 (34.9)	60 (31.8)	201 (28.0)	
Living alone (yes)	14 (6.9)	44 (11.7)	29 (15.3)	58 (8.1)	0.008
Body-mass index (kg/m^2^)					
Underweight (< 18.5)	9 (4.4)	55 (14.7)	11 (5.8)	52 (7.3)	< 0.001
Ideal weight (18.5 ~ 22.9)	82 (40.4)	155 (41.3)	75 (39.7)	264 (36.8)	
Overweight (23 ~ 24.9)	46 (22.7)	75 (20.0)	42 (22.2)	142 (19.8)	
Obese (≥25)	66 (32.5)	90 (24.0)	61 (32.3)	259 (36.1)	
Current smoker (yes)	2 (1.0)	11 (2.9)	1 (0.5)	11 (1.5)	0.129
Regular alcohol consumption (≥3 times per week)	2 (1.0)	8 (2.1)	15 (7.9)	15 (2.1)	< 0.001
Regular exercise (≥3 times per week)	123 (60.6)	171 (45.6)	103 (54.5)	320 (44.6)	< 0.001
Bone mineral density (T-score < − 2.5)	47 (23.2)	201 (53.6)	108 (57.1)	149 (20.8)	< 0.001
Fall history in the past year (no.)					
0	170 (83.7)	261 (69.6)	125 (66.1)	485 (67.6)	0.001
1	17 (8.4)	54 (14.4)	33 (17.5)	104 (14.5)	
≥ 2	16 (7.9)	60 (16.0)	31 (16.4)	128 (17.9)	
Previous fractures since the age of 50 years (yes)	28 (13.8)	110 (29.3)	53 (28.0)	92 (12.8)	< 0.001
Number of chronic conditions (medium (range))	2.0 (0 ~ 8)	3.0 (0 ~ 10)	3.0 (0 ~ 11)	2.0 (0 ~ 9)	< 0.001
Medication use					
Any medication use	133 (65.5)	325 (86.7)	155 (82.0)	593 (82.7)	< 0.001
Antihypertensive drugs	74 (36.5)	177 (47.2)	84 (44.4)	315 (43.9)	0.114
Sedatives/hypnotics	15 (7.4)	64 (17.1)	32 (16.9)	78 (10.9)	0.001
Antidiabetics	30 (14.8)	109 (29.1)	53 (28.0)	167 (23.3)	0.001
Vitamins	25 (12.3)	44 (11.7)	31 (16.4)	140 (19.5)	0.295
Calcium	30 (14.8)	56 (14.9)	38 (20.1)	148 (20.6)	0.530
Antihistamines	5 (2.5)	18 (4.8)	5 (2.6)	15 (2.1)	0.591
Visual acuity					
Impaired	47 (23.1)	158 (42.1)	83 (43.9)	163 (22.7)	< 0.001
Good	156 (76.9)	217 (57.9)	106 (56.1)	554 (77.3)	
Use of walking aids preinjury (yes)	25 (12.3)	105 (28.0)	35 (18.5)	159 (22.2)	< 0.001
Cognitive impairment (SPMSQ ≥5)^α^	13 (6.4)	77 (20.5)	24 (12.7)	91 (12.7)	< 0.001
Depressive symptoms (GDS > 5)^α^	9 (4.4)	39 (10.4)	15 (7.9)	26 (3.6)	< 0.001
Fear of falling (medium (range)) (points)	0.5 (0 ~ 10)	1.5 (0 ~ 10)	2.0 (0 ~ 10)	2.0 (0 ~ 10)	0.188

^α^ *GDS* Geriatric Depression Scale, *SD* standard deviation, *SPMSQ* Short Portable Mental Status Questionnaire

Distributions of situational factors in the three fracture groups and controls are shown in Table 2. Among the four groups, significant differences were detected in the location, mode and direction of the fall, the presence of an uneven floor, and hitting an object and initiating a protective response during the fall. Among these groups, distal-forearm fractures tended to have occurred more frequently during outdoor activities, tripping, and falling forward, and with an uneven floor. Vertebral fractures tended to have occurred more frequently during a step-down, when falling backwards, and with a stable COM. Hip fractures tended to have occurred more frequently during outdoor activities, when leg-weakness occurred, when stepping-down, falling in a sideways direction, and in the absence of a protective response.

**Table 2:** Distributions of situational factors among four groups of distal-forearm fracture, hip fracture, vertebral fracture, and soft-tissue injury

Characteristic	Distal-forearm fracture (*N* = 203)	Hip fracture (*N* = 375)	Vertebral fracture (*N* = 189)	Soft-tissue injury (*N* = 717)	*p*
	*n* (%)	*n* (%)	*n* (%)	*n* (%)	
Injury location					
Indoors	108 (53.2)	293 (78.1)	140 (74.1)	510 (71.1)	< 0.001
Outdoors	95 (46.8)	82 (21.9)	49 (25.9)	207 (28.9)	
Activity during the fall					
Toileting	20 (9.9)	50 (13.3)	25 (13.2)	102 (14.2)	0.108
Get in/out of bed	11 (5.4)	58 (15.5)	30 (15.9)	74 (10.3)	
Negotiating stairs	22 (10.8)	23 (6.1)	14 (7.4)	78 (10.9)	
Doing housework	25 (12.3)	36 (9.6)	33 (17.4)	73 (10.2)	
Walking	109 (53.7)	181 (48.3)	71 (37.6)	336 (46.9)	
Other	16 (7.9)	27 (7.2)	16 (8.5)	54 (7.5)	
Fall mode					
Slipping	82 (40.4)	51 (13.6)	45 (23.8)	115 (16.0)	< 0.001
Tripping	39 (19.2)	49 (13.0)	29 (15.3)	135 (18.8)	
Leg-weakness	10 (4.9)	75 (20.0)	18 (9.5)	118 (16.5)	
Fainting	7 (3.5)	64 (17.1)	23 (12.2)	132 (18.4)	
Step-down	65 (32.0)	136 (36.3)	74 (39.2)	217 (30.3)	
Fall direction					
Forwards	95 (46.8)	57 (15.2)	49 (25.9)	341 (47.6)	< 0.001
Backwards	52 (25.6)	169 (45.1)	101 (53.5)	267 (37.2)	
Sideways	56 (27.6)	149 (39.7)	39 (20.6)	109 (15.2)	
Change in center of mass					
Stable	24 (11.8)	62 (16.5)	44 (23.3)	87 (12.1)	0.125
Vertical change	25 (12.3)	52 (13.9)	24 (12.7)	90 (12.6)	
Horizontal change	122 (60.1)	180 (48.0)	77 (40.7)	387 (54.0)	
Both changes	32 (15.8)	81 (21.6)	44 (23.3)	153 (21.3)	
Uneven floor (yes)	103 (50.7)	59 (15.7)	41 (21.7)	157 (21.9)	< 0.001
Hitting an object during the fall (yes)	18 (8.9)	25 (6.7)	25 (13.2)	121 (16.9)	0.001
Protective response during the fall (yes)	25 (12.3)	54 (14.4)	19 (10.1)	69 (9.6)	0.112

Table 3 shows results of the multivariable multinomial logistic regression analysis for distal-forearm fractures, hip fractures, and vertebral fractures compared to soft-tissue injuries. After adjusting for individual characteristics (age, educational level, living arrangement, BMI, BMD, and number of chronic conditions), compared to soft-tissue injuries, distal-forearm fractures were significantly more likely to occur in slips (OR = 11.0; 95% CI = 4.76 ~ 25.4), trips (OR = 3.40; 95% CI = 1.42 ~ 8.17), step-downs (OR = 4.95; 95% CI = 2.15 ~ 11.4) and from sideways falls (OR = 1.73; 95% CI = 1.12 ~ 2.67) and significantly less likely to occur in indoor activities (OR = 0.62; 95% CI = 0.42 ~ 0.90) or from backwards falls (OR = 0.62; 95% CI = 0.41 ~ 0.95). Hip fractures were significantly more likely to occur in step-downs (OR = 1.76; 95% CI = 1.13 ~ 2.75) and from backwards (OR = 3.16; 95% CI = 2.15 ~ 4.64) and sideways falls (OR = 5.56; 95% CI = 3.67 ~ 8.41) and significantly less likely to when hitting an object (OR = 0.26; 95% CI = 0.13 ~ 0.52) or initiating a protective response (OR = 0.58; 95% CI = 0.36 ~ 0.93) during the fall. Vertebral fractures were significantly more likely to occur in slips (OR = 2.42; 95% CI = 1.30 ~ 4.50) and step-downs (OR = 2.53; 95% CI = 1.43 ~ 4.48) and from backwards falls (OR = 2.15; 95% CI = 1.39 ~ 3.32). For model checking, *p* values of the Hosmer-Lemeshow test ranged from 0.569 to 0.912, indicating no significant differences between observed and predicted values.

**Table 3:** Results of the multinomial logistic regression analysis: adjusted odds ratios (ORs) and 95% confidence intervals (CIs) of situational factors for distal-forearm fracture, hip fracture, and vertebral fracture, respectively, compared to a soft-tissue injury ^a^

Characteristic	Distal-forearm fracture		Hip fracture		Vertebral fracture	
	OR (95% CI)	*p*	OR (95% CI)	*p*	OR (95% CI)	*p*
Injury location						
Outdoors	1.00		1.00		1.00	
Indoors	0.62 (0.42, 0.90)	0.012	1.28 (0.89, 1.83)	0.205	1.40 (0.91, 2.15)	0.111
Fall mode						
Fainting	1.00		1.00		1.00	
Slipping	11.0 (4.76, 25.4)	< 0.001	1.29 (0.77, 2.17)	0.335	2.42 (1.30, 4.50)	0.006
Tripping	3.40 (1.42, 8.17)	0.006	1.35 (0.78, 2.34)	0.286	1.79 (0.91, 3.54)	0.094
Leg-weakness	1.94 (0.70, 5.35)	0.202	1.27 (0.77, 2.07)	0.348	0.94 (0.46, 1.94)	0.870
Step-down	4.95 (2.15, 11.4)	< 0.001	1.76 (1.13, 2.75)	0.012	2.53 (1.43, 4.48)	0.001
Fall direction						
Forwards	1.00		1.00		1.00	
Backwards	0.62 (0.41, 0.95)	0.027	3.16 (2.15, 4.64)	< 0.001	2.15 (1.39, 3.32)	0.001
Sideways	1.73 (1.12, 2.67)	0.014	5.56 (3.67, 8.41)	< 0.001	1.49 (0.89, 2.51)	0.134
Hitting an object during the fall	0.67 (0.36, 1.26)	0.211	0.26 (0.13, 0.52)	< 0.001	0.75 (0.35, 1.60)	0.455
Protective response during the fall	0.72 (0.42, 1.25)	0.245	0.58 (0.36, 0.93)	0.022	0.98 (0.54, 1.79)	0.950

^a^ All models were adjusted for age at injury, educational level, body-mass index, bone mineral density, and the number of chronic conditions

Table 4 shows the validation results of the binary logistic regression analyses separately for distal-forearm fractures, hip fractures, and vertebral fractures. With few exceptions (i.e., sideways falls for distal-forearm fractures, step-downs for hip fractures, and slipping for vertebral fractures), most of associations between situational factors and each type of fracture compared to the other two types of fracture, despite being weakened to some extent, were similar to results when soft-tissue injuries were used as the control group. For instance, the adjusted OR of falling backwards changed to 0.46 (95% CI = 0.30 ~ 0.69) for distal-forearm fractures, 2.75 (95% CI = 1.79 ~ 3.97) for hip fractures, and 1.70 (95% CI = 1.12 ~ 2.57) for vertebral fractures.

**Table 4:** Validation results of three binary logistic regression analyses with adjusted odds ratios (ORs) and 95% confidence intervals (CIs) of situational factors separately for each of distal-forearm fracture, hip fracture, and vertebral fracture, respectively, compared to the other types of fracture ^a^

Characteristic	Distal-forearm fracture		Hip fracture		Vertebral fracture	
	OR (95% CI)	*p*	OR (95% CI)	*p*	OR (95% CI)	*p*
Injury location						
Outdoors	1.00		1.00		1.00	
Indoors	0.56 (0.39, 0.80)	0.001	1.25 (0.90, 1.75)	0.187	1.43 (0.96, 2.13)	0.076
Fall mode						
Fainting	1.00		1.00		1.00	
Slipping	8.66 (3.81, 19.7)	< 0.001	0.73 (0.45, 1.17)	0.191	1.60 (0.89, 2.85)	0.114
Tripping	2.74 (1.16, 6.52)	0.022	1.06 (0.64, 1.76)	0.820	1.53 (0.81, 2.89)	0.189
Leg-weakness	1.79 (0.66, 4.88)	0.253	1.18 (0.75, 1.87)	0.477	0.85 (0.43, 1.68)	0.630
Step-down	3.76 (1.66, 8.53)	0.002	1.16 (0.77, 1.75)	0.468	1.84 (1.08, 3.14)	0.025
Fall direction						
Forwards	1.00		1.00		1.00	
Backwards	0.46 (0.30, 0.69)	< 0.001	2.75 (1.79, 3.97)	< 0.001	1.70 (1.12, 2.57)	0.013
Sideways	1.11 (0.74, 1.68)	0.615	4.63 (3.16, 6.78)	< 0.001	0.75 (0.46, 1.22)	0.239
Hitting an object during the fall	0.83 (0.44, 1.54)	0.546	0.29 (0.15, 0.56)	< 0.001	0.98 (0.47, 2.07)	0.965
Protective response during the fall	0.82 (0.49, 1.37)	0.451	0.61 (0.40, 0.93)	0.022	1.32 (0.76, 2.29)	0.323

^a^ All models were adjusted for age at injury, educational level, body-mass index, bone mineral density, and the number of chronic conditions

## Discussion

Previous studies reported that initial distal forearm or vertebral fractures were associated with subsequent fractures of the vertebrae, hip, distal forearm, and other sites, and risks of different fracture types resulted from similar individual characteristics such as an older age and low BMD [16, 17, 25, 26]. This study compared the relative impacts of situational factors during a fall on the three common types of fracture in older women, while controlling for individual characteristics. As a result, large variations in situational risk factors, such as location, mode, and direction of falls, existed among the three fracture types of the distal forearm, vertebrae, and hip in older women.

In contrast to distal-forearm fractures associated with falls in the forward and sideways directions in the study, two studies of older women reported that distal-forearm fractures were more likely to have occurred when they fell backwards or sideways, compared to those with no fracture [11, 12]. One explanation for the differences might be that fall modes that were not evaluated in the two studies could have confounded their findings, since slipping falls are usually concomitant with a backward fall direction. Alternatively, our participants who had lower BMIs than subjects in those two studies (mean BMI: 19.5 vs. 26.0 and 25.9 kg/m^2^) may have had increased chances to walk with a faster gait speed, thereby providing more opportunities to have a forward loss of balance; overweight and obese older people tend to walk at a slower speed and exhibit poorer functional fitness and mobility compared to those of a normal weight [27, 28]. Furthermore, stepping-down falls in our study were associated with increased risks of all the three types of fractures. It is possible that as the body COM descends to a lower level, failure to transform higher potential energy and control the relevant momentum causes a higher-impact fall with a more-serious injury, particularly during unexpected height changes (e.g., from a street curb or an uneven floor, into a hole, or during stair descent) [29].

Consistent with previous studies [8, 13, 30, 31], hip fractures were strongly associated with falling sideways. Some researchers suggested that people may execute backward rotation during a fall to impact the buttocks or execute forward rotation to impact the outstretched hands to avoid an impact to the hip with an initial sideways fall direction [32]. However, converting a sideways fall into a backwards one should be done with caution because falling backwards has also been linked with the incidence and severity of traumatic brain injuries [33, 34]; moreover, falling backwards also increases the risks of hip and vertebral fractures. Safer landing strategies based on falling directions to reduce fall severity and prevent serious injuries were recently explored in detail [35]. In addition to the fall direction, consequences of falls might also depend on the velocity and force of impact and the protective reaction time [36]. A low-impact sideways fall might result in a minor injury or a wrist fracture, whereas a fast, high-impact sideways fall might result in a hip fracture, particularly for frail older persons who may have too-late compensatory reactions (e.g., stepping and grasping) in response to a loss of balance [37]. Older women not only have lower BMDs but also less effectively provide arm protection reactions in preventing falling to the ground compared to older men [13, 38]. It should also be noted that sufficient muscle strength of the extremities is essential for successfully conducting safe landing strategies and effective compensatory reactions. The association of hitting an object during a fall with hip fractures might reflect competing risks of non-hip injuries and hip fractures during a fall, where, for instance, hitting the arms or hands first might lead to an injury to the forearm but prevent a direct impact to the hip [39].

Although individual characteristics of age and BMD were often reported as strong personal risk factors for older women [40], probably because women have lower vertebral strength and greater declines in strength over time compared to men [41], one-half of all clinical vertebral fractures occur in persons without a diagnosis of osteoporosis [42]. In this study, vertebral fractures occurred most frequently in a backwards direction and the step-down mode, while backwards and step-down falls also exhibited higher risks of vertebral fractures compared to other directions or modes. It is difficult to make comparisons with prior studies because no study has provided situational factors during falls resulting in vertebral fractures, even though some biomechanical studies simulated backward falls to evaluate impact forces on the lumbosacral spine [43].

There are several limitations to this study. First, our participants might not be representative of a population-based sample for the three types of fractures and soft-tissue injuries. Almost all of the new distal-forearm and hip fractures were identified in the ED, while in contrast, a portion of vertebral fractures and soft-tissue injuries might not have been serious or painful enough for an older person to have sought ED services. Nevertheless, our validation analyses in which patients who sustained the other two types of fracture were used as the comparison group produced similar results. Second, the results cannot be generalized to all older fallers, particularly those who are mentally and physically dependent, because not all older fallers come to the ED and some were excluded from the study due to severe cognitive impairment, non-ambulation, or being medically incapacitated. Third, self-reported data on situational factors were difficult to validate, even though our method of immediate assessment at the ED may have reduced memory lapses and recall errors related to time factors to some degree. Differential memory lapses and recall errors between older subjects and proxy respondents might also exist. Fourth, several medications related to risks of falls and fractures (e.g., antipsychotics, antidepressants, antiepileptics, anti-Parkinson drugs, opioids, and proton pump inhibitors) that might have confounded the results were not measured or assessed in the study. Finally, the impact velocity and force which affect the fall severity were not measured, and their distributions might not be the same among the three fracture types.

## Conclusions

Despite the benefits of primary fall-prevention programs (e.g., multifactorial interventions and exercise training), individuals who participate in these programs still fall. Our study demonstrated that large variations in situational risk factors, such as location, mode, and direction of the fall, may exist among fractures of the distal forearm, hip, and vertebrae in older women. A combination of personal and situational risk factors may display a more-comprehensive risk profile for each type of fracture, and an intervention that adds training programs on fall safety, such as safe landing strategies and effective compensatory reactions, can be valuable in preventing serious injuries due to a fall.

## Supplementary Information

**Additional file 1: Supplementary materials.** Full Questionnaire in English version.

## Data Availability

The datasets generated and analyzed during the current study are not publicly available due to ethical restrictions and patient confidentiality but are available from the corresponding author upon reasonable request.

## Abbreviations

BMD: Bone mineral density
BMI: Body-mass index
COM: Center of mass
ED: Emergency department
GDS: Geriatric Depression Scale
SPMSQ: Short Portable Mental Status Questionnaire
SD: Standard deviation
