# Supplementary material for: Variations in situational risk factors for fractures of the distal forearm, hip, and vertebrae in older women
Source: BMC Geriatr. 2021 Mar 31;21:214. doi: 10.1186/s12877-021-02157-2 (PMC8011116; doi:10.1186/s12877-021-02157-2)
Supplement: Supplementary file 1 — Additional file 1: Supplementary materials. Full Questionnaire in English version. [file 12877_2021_2157_MOESM1_ESM.doc]

**Supplementary materials:** **Full Questionnaire in English version**

**Title:** Variations in Situational Risk Factors for Fractures of the Distal Forearm, Hip, and Vertebrae in Older Women

**Authors:** Wen-Yu Yu^1,2^, MD; Hei-Fen Hwang^3^, PhD; and Mau-Roung Lin^2^, PhD

**Affiliations:**

^1^Department of Emergency Medicine, Taipei Medical University Hospital, Taipei, Taiwan, R.O.C.

^2^Institute of Injury Prevention and Control, College of Public Health, Taipei Medical University, Taipei, Taiwan, R.O.C.

^3^Department of Nursing, National Taipei University of Nursing and Health Sciences, Taipei, Taiwan, R.O.C.

Questionnaire for fall-related information Questionnaire No.

Date of registration: YYYY/MM/DD

Hospital name:

Name: Date of Injury: YYYY/MM/DD

Sex: □male □female

Year of birth: YYYY

Body Height: cm;

Body Weight: Kg

1. Sociodemographic and lifestyles:
2. Living arrangement: ➀□living in agency ➁□living at home, number of family members
3. Marital status: ➀□Single➁□Married➂□Widowed➃□Divorced➄□Others
4. Educational level (graduation): ➀□college or above ➁□Senior high ➂□Junior high

➃□Elementary school ➄□Illiterate

1. Current smoking: ➀□No ➁□Yes
2. Regular alcohol consumption: ➀□No ➁□Yes【at least 3 times per week)】
3. Regular exercise: ➀□No ➁□Yes【at least 3 times per week)】
4. Medical characteristics:
5. Did you have a fall in the past year?

➀□No ➁□Yes, times,

In which seeking medical care times; In which need a hospitalization times

1. Did you have a fracture history since age of 50 years?

➀□No ➁□Yes, fractures site and age at injury

1. Currently, do you have any chronic disease? □No
2. Cardiovascular diseases: □Hypertension □ Heart disease □Stroke
3. Endocrine system: □Diabetes mellitus
4. Respiratory tract disease: □Asthma □Chronic obstructive pulmonary disease □Tuberculosis

(4)Others: □Malignant tumors □Gastric disease □Renal disease □Degenerative arthritis □Cataracts

□Alzheimer’s disease □Parkinson’s disease □Others

1. Regular use of medications at present: □No □Yes, which medication?

Antihypertensive drugs: □No □Yes,

Antidiabetics (include insulin): □No □Yes,

Calcium: □No □Yes Vitamins: □No □Yes,

Antihistamine: □No □Yes, Prostate hypertrophy drugs: □No □Yes,

Sedatives / hypnotics: □No □Yes,

Others: □No □Yes,

1. Bone Mineral Density value : T-score:

(Check medical records after the DEXA measurement)

1. Situational exposures during the fall
2. Whether to use walking aids before falling? □No □Yes
3. Wat was the floor condition of the fall? □No abnormal shape □Waxing □Stagnant water □Uneven □Other_________
4. What was the location of the fall? □Indoors □Outdoors
5. What was the activity during the fall: □Toileting □Get in/out of bed □Negotiating stairs □Doing housework □Walking □Other________
6. What was the posture during the fall? □Keep standing □Keep sitting □Keep squatting

□Sitting changing to standing □Standing changing to sitting □Squatting to standing

□Standing to squatting □Squatting to sitting □Sitting to squatting □Turn around □Bend forward □Bend backward □Horizontal jump □Vertical jump □Normal speed walking □Quick walking

□Jogging □Quick running □Push your toes □Other ______

1. What was the mode of the fall? □Slipping □Tripping □Leg-weakness □Fainting □Step-down
2. What was the fall direction? □Forward □Backward □Sideways
3. Had your body been hit during the fall: □No □Yes
4. Did you initiate protective response during the fall: □No □Yes
5. Any injuries due to the fall? (Check medical chart)
6. Bruises and bruises: □Head □Face □Neck □Chest □Abdomen □Upper back □Lower back

□Upper limbs □Lower limbs (including buttocks) □Other______

1. Laceration: □Head □Face □Neck □Chest □Abdomen □Upper back □Lower back

□Upper limbs □Lower limbs □Other______

1. Strain or sprain: □Neck □Elbow □Wrist □Waist □Knee □Ankle □Other______
2. Joint capsule rupture: □Neck □Elbow □Wrist □Waist □Knee □Ankle □Other______
3. Fracture site:

□head【□frontal bone □parietal bone □temporal bone □occipital bone □other 】

□Face【□Nasal bone □Cheek bone □Upper jaw □Mandible □Other 】

□Chest【□Clavicle □Sternum □Rib: left rib; right rib □Other 】

□Upper limbs 【□Scapula □Humerus □Ulna □Radius □Carpal bones □Metacarpal bones

□Phalanges □Other 】

□Lower limbs【□Femur □Patina □Tibia □Fibula □Metatarsal bones □Phalanges □Other 】

□Pelvis【□Ilium □Ischia □Pubic □Sacral spine □Caudal spine □Other 】

□Spine【□Cervical spine section □Thoracic spine section □Lumbar spine section】

14. Other diagnosis

1. Functional abilities:
2. Consisting of visual acuity: Left Right [use Rosenbaum card]
3. Cognitive status
4. How old are you?
5. What is the date today? □Year/ □Month/ □Date / □Week
6. Is here a hospital (clinic), activity center or home? (□2, respond naturally; □1, respond after reminding)
7. ➀ What is 20 minus 3?

➁ Minus 3, What is it?

➂ Minus 3, What is it?

➃ Minus 3, What is it?

1. What is your postal address (Name out any of the counties, towns, and streets)
2. What is your mother's surname? (When the respondent make sure the answer being correct)
3. Who is the current president? (When the respondent can recognize the answer being correct)
4. Who was the last president?
5. When were you born?

➀＿＿＿Year (If the zodiac is correct, it will be considered correct, get 1 point)

➁＿＿＿month

➂＿＿＿day

1. Depressive symptoms
2. Are you basically satisfied with your life? ➀ □Yes ➁ □No
3. Have you dropped many of your activities and interests? ➀ □Yes ➁ □No
4. Do you feel that your life is empty? ➀ □Yes ➁ □No
5. Do you often get bored? ➀ □Yes ➁ □No
6. Are you in good spirits most of the time? ➀ □Yes ➁ □No
7. Are you afraid that something bad is going to happen to you? ➀ □Yes ➁ □No
8. Do you feel happy most of the time? ➀ □Yes ➁ □No
9. Do you often feel helpless? ➀ □Yes ➁ □No
10. Do you prefer to stay at home, rather than going out and doing new things? ➀ □Yes ➁ □No
11. Do you feel you have more problems with memory than most? ➀ □Yes ➁ □No
12. Do you think it is wonderful to be alive now? ➀ □Yes ➁ □No
13. Do you feel pretty worthless the way you are now? ➀ □Yes ➁ □No
14. Do you feel full of energy? ➀ □Yes ➁ □No
15. Do you feel that your situation is hopeless? ➀ □Yes ➁ □No
16. Do you think that most people are better off than you are? ➀ □Yes ➁ □No
17. Fear of falling

How worried about falling before falling? ➀□Not worried at all ➁□A little worried ➂□Moderately worried ➃□Very worried ➄□Extremely worried
